# Supplementary material for: Neuronal let-7b-5p acts through the Hippo-YAP pathway in neonatal encephalopathy
Source: Commun Biol. 2021 Sep 30;4:1143. doi: 10.1038/s42003-021-02672-3 (PMC8484486; doi:10.1038/s42003-021-02672-3)

Supplementary information:

Supplementary Table 1. Clinical characteristics of neonates whose samples were analysed by microRNA Next Generation Sequencing

All data are median (range) unless otherwise indicated. NE, neonatal encephalopathy; TH, therapeutic hypothermia.

**Perinatal characteristics**	**Moderate to severe NE with TH - unfavourable outcome n = 4**	**Moderate to severe NE with TH - favourable outcome n = 4**	**Mild NE with No TH n = 4**	**Test Control neonates n = 4**
Gestational age, (completed weeks + days)	39+4 (38+6 to 42+4)	37+4 (35+6 to 41+1)	39+5 (37+2 to 41+4)	38+4 (37+2 to 39+1)
Male sex, n (%)	3 (75%)	4 (100%)	1 (25%)	3 (75%)
Birth weight (g)	3880 (2920 - 4262)	2820 (2200 - 3569)	3145 (2380 - 4045)	3190 (2730 - 3610)
Apgar score at 10 min	4 (2 - 6)	5 (0 -7)	9 (5 -10)	10 (9 -10)
Worst pH within 1 h	6.93 (6.56 - 7.25)	6.89 (6.63 - 7.30)	6.94 (6.91 - 6.97)	n/a
Worst base deficit within 1 h	-15.7 (-35 to -6.4)	-19.4 (-25.7 to -12)	-11.6 (-20.9 to -10.7)	n/a
Need for respiratory support at 10 min age, n (%)	2 (50%)	3 (75%)	1 (25%)	0 (0%)
Need for chest compressions, n (%)	2 (50%)	3 (75%)	0 (0%)	0 (0%)

Supplementary Fig. 1. Grouping of neonates according to their characteristics along with details of samples collected. NE, neonatal encephalopathy; TH, therapeutic hypothermia.

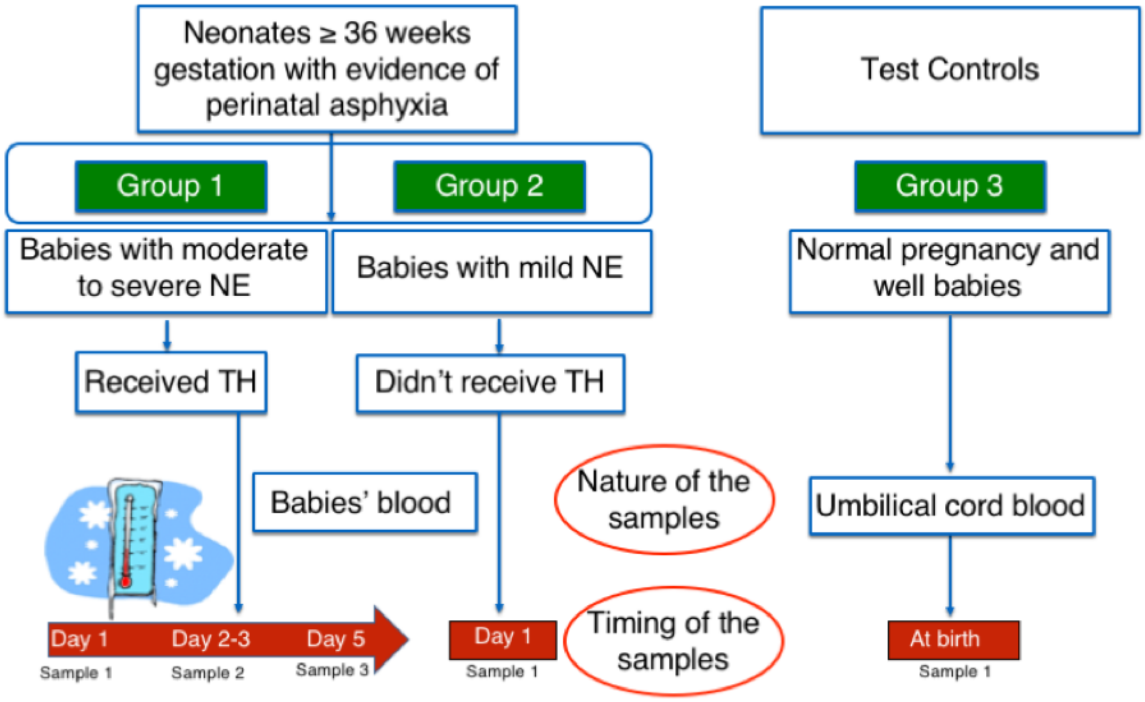

Supplementary Fig. 2. Heatmap of pathways union from KEGG analysis for mild vs moderate to severe NE with favourable outcome

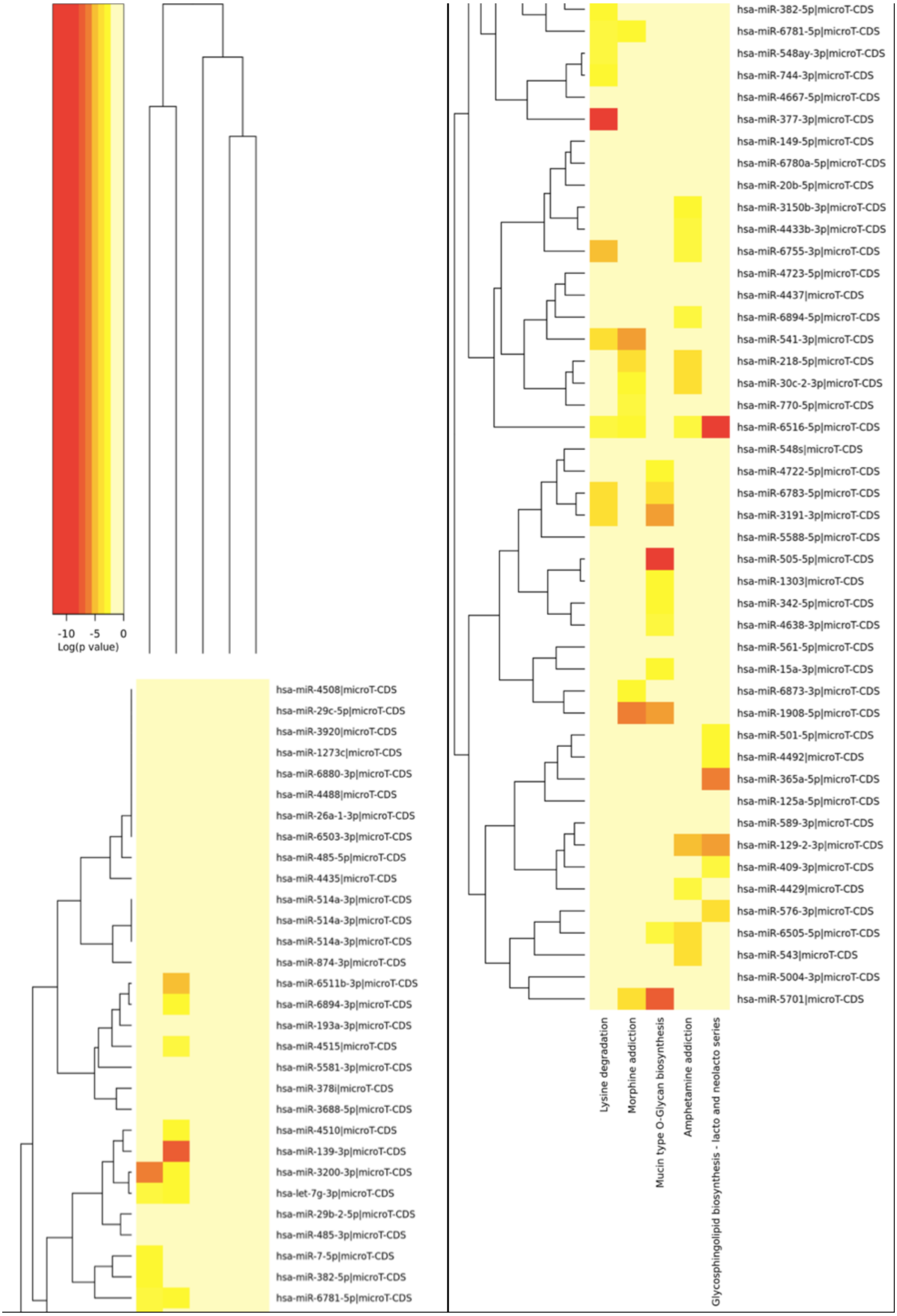

Supplementary Fig. 3. Heatmap of pathways union from KEGG analysis for mild vs moderate to severe NE with unfavourable outcome

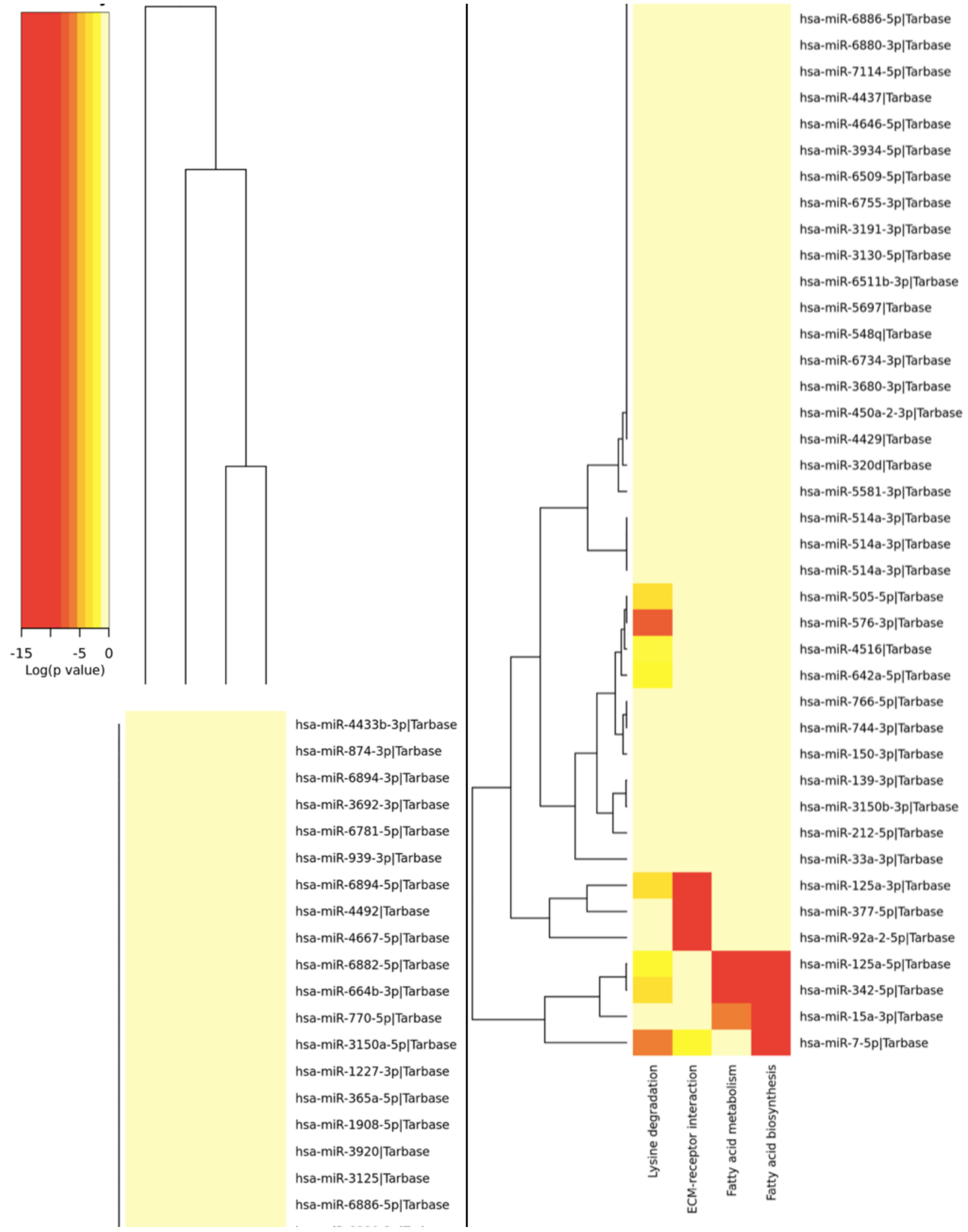

Supplementary Fig. 4. Heatmap of pathways union from KEGG analysis for moderate to severe NE with favourable vs unfavourable outcome

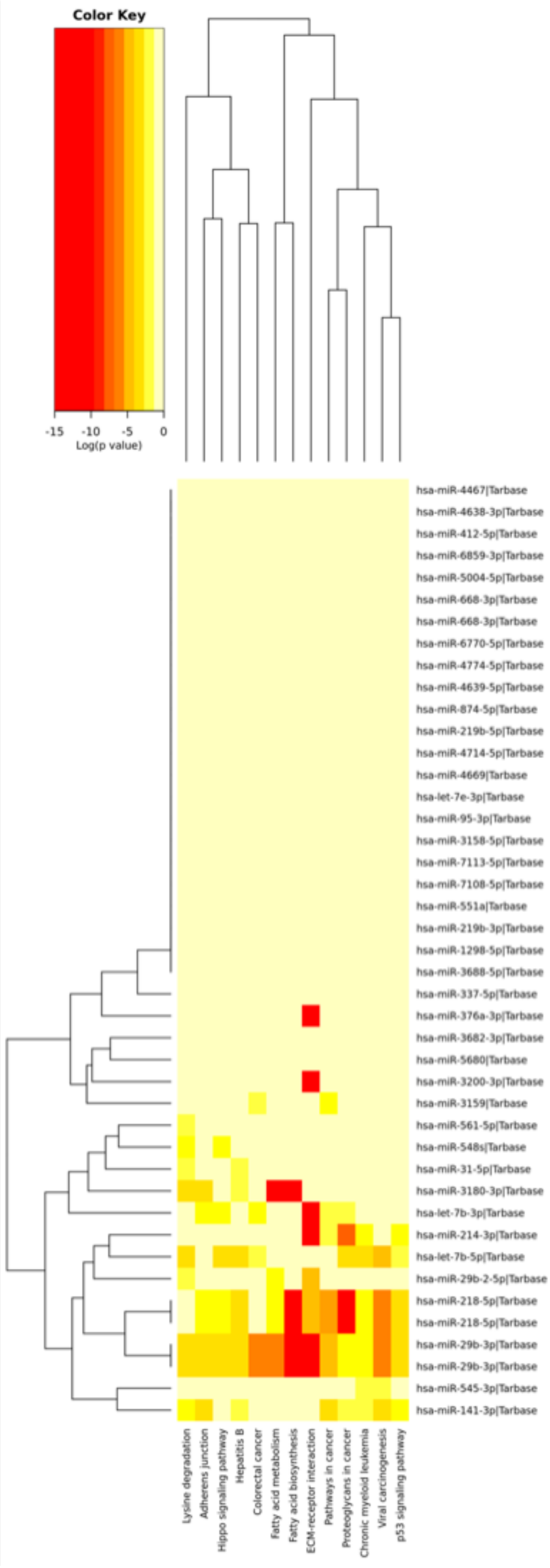

Supplement: Supplementary file 2 — Supplemental Material [file 42003_2021_2672_MOESM2_ESM.pdf]
