# Supplementary material for: Neuronal let-7b-5p acts through the Hippo-YAP pathway in neonatal encephalopathy
Source: Commun Biol. 2021 Sep 30;4:1143. doi: 10.1038/s42003-021-02672-3 (PMC8484486; doi:10.1038/s42003-021-02672-3)
Supplement: Supplementary file 3 — Description of Additional Supplementary Files [file 42003_2021_2672_MOESM3_ESM.pdf]

Description of Additional Supplementary Files

File name: Supplementary Data 1

Description: Validation data – miRNA Neonates.

File name: Supplementary Data 2

Description: Cell cultures data.

File name: Supplementary Data 3

Description: Murine let-7b-5p data.
