# Supplementary material for: Neuronal let-7b-5p acts through the Hippo-YAP pathway in neonatal encephalopathy
Source: Commun Biol. 2021 Sep 30;4:1143. doi: 10.1038/s42003-021-02672-3 (PMC8484486; doi:10.1038/s42003-021-02672-3)
Supplement: Supplementary file 7 — Reporting Summary [file 42003_2021_2672_MOESM7_ESM.pdf]

nature research

Corresponding author(s): Dr Vennila Ponnusamy

Last updated by author(s): Jul 31, 2021

Reporting Summary

Nature Research wishes to improve the reproducibility of the work that we publish. This form provides structure for consistency and transparency in reporting. For further information on Nature Research policies, see our Editorial Policies and the Editorial Policy Checklist.

Statistics

For all statistical analyses, confirm that the following items are present in the figure legend, table legend, main text, or Methods section.

n/a	Confirmed	
☐	☒	The exact sample size (*n*) for each experimental group/condition, given as a discrete number and unit of measurement
☐	☒	A statement on whether measurements were taken from distinct samples or whether the same sample was measured repeatedly
☐	☒	The statistical test(s) used AND whether they are one- or two-sided<br>*Only common tests should be described solely by name; describe more complex techniques in the Methods section.*
☒	☐	A description of all covariates tested
☐	☒	A description of any assumptions or corrections, such as tests of normality and adjustment for multiple comparisons
☐	☒	A full description of the statistical parameters including central tendency (e.g. means) or other basic estimates (e.g. regression coefficient) AND variation (e.g. standard deviation) or associated estimates of uncertainty (e.g. confidence intervals)
☒	☐	For null hypothesis testing, the test statistic (e.g. *F*, *t*, *r*) with confidence intervals, effect sizes, degrees of freedom and *P* value noted<br>*Give P values as exact values whenever suitable.*
☒	☐	For Bayesian analysis, information on the choice of priors and Markov chain Monte Carlo settings
☒	☐	For hierarchical and complex designs, identification of the appropriate level for tests and full reporting of outcomes
☒	☐	Estimates of effect sizes (e.g. Cohen's *d*, Pearson's *r*), indicating how they were calculated

Our web collection on statistics for biologists contains articles on many of the points above.

Software and code

Policy information about availability of computer code

Data collection	Images were obtained using ImageJ (1.51v) with customised plugins including 'Channel merging', 'Intensity and Co-localisation analysis' and "Cell count" macros.
Data analysis	Bioinformatics analysis for miRNA NGS data was performed using Partek® Genomics Suite® software Version 7. MiRNA pathway analysis was performed using mirPath V.3 to predict miRNA targets. Hierarchical clustering was performed using R program. All other data analysis was performed using GraphPad Prism 8.

For manuscripts utilizing custom algorithms or software that are central to the research but not yet described in published literature, software must be made available to editors and reviewers. We strongly encourage code deposition in a community repository (e.g. GitHub). See the Nature Research guidelines for submitting code & software for further information.

Data

Policy information about availability of data

All manuscripts must include a data availability statement. This statement should provide the following information, where applicable:
- Accession codes, unique identifiers, or web links for publicly available datasets
- A list of figures that have associated raw data
- A description of any restrictions on data availability

All miRNA next generation sequencing data files have been deposited in the Gene Expression Omnibus (www.ncbi.nlm.nih.gov/geo/) (accession number(s) GSE181127), and are available for public from 5th August 2021, Link here: https://www.ncbi.nlm.nih.gov/geo/query/acc.cgi?acc=GSE181127)

Field-specific reporting

Please select the one below that is the best fit for your research. If you are not sure, read the appropriate sections before making your selection.

[x] Life sciences [ ] Behavioural & social sciences [ ] Ecological, evolutionary & environmental sciences

For a reference copy of the document with all sections, see nature.com/documents/nr-reporting-summary-flat.pdf

Life sciences study design

All studies must disclose on these points even when the disclosure is negative.

Sample size	Based on the prediction of generating around 20 million reads per sample, a minimum sample size of 20 samples was estimated for miRNA NGS analysis. A cohort of 16 neonates was included, with four per subgroup selected on the basis of robust clinical grouping and MRI outcome to provide a total of 32 samples at the three time points.
Data exclusions	No data was excluded
Replication	Validation of selected miRNAs of significance from miRNA next generation sequencing was performed using quantitative RT-qPCR
Randomization	Consecutive babies enrolled in a multi-centre biomaker study called BIBINs were included in this work, if adequate samples were available at all time points.
Blinding	Blinding is not relevant to this study as it is an exploratory study on miRNAs.

Reporting for specific materials, systems and methods

We require information from authors about some types of materials, experimental systems and methods used in many studies. Here, indicate whether each material, system or method listed is relevant to your study. If you are not sure if a list item applies to your research, read the appropriate section before selecting a response.

Materials & experimental systems

n/a	Involved in the study	
[ ]	[x]	Antibodies
[x]	[ ]	Eukaryotic cell lines
[x]	[ ]	Palaeontology and archaeology
[ ]	[x]	Animals and other organisms
[ ]	[x]	Human research participants
[x]	[ ]	Clinical data
[x]	[ ]	Dual use research of concern

Methods

n/a	Involved in the study	
[x]	[ ]	ChIP-seq
[x]	[ ]	Flow cytometry
[x]	[ ]	MRI-based neuroimaging

Antibodies

Antibodies used	Mouse anti-NeuN (1:500, MAB377, Merck Millipore) ; rabbit anti-cleaved caspase 3 (1:100, Cat No. 9664, Cell Signaling Technology); rabbit anti-YAP (1:200, Cat No. 14074S, Cell Signaling Technology)
Validation	https://www.merckmillipore.com/GB/en/product/Anti-NeuN-Antibody-clone-A60,MM_NF-MAB377<br>https://www.cellsignal.com//products/primary-antibodies/cleaved-caspase-3-asp175-5a1e-rabbit-mab/9664<br>https://www.cellsignal.com/products/primary-antibodies/yap-d8h1x-xp-rabbit-mab/14074?site-search-type=Products&N=4294956287&Ntt=14074s&fromPage=plp&_requestid=2684383

Animals and other organisms

Policy information about studies involving animals; ARRIVE guidelines recommended for reporting animal research

Laboratory animals	Time-pregnant Wistar rats of both sexes were purchased from CERJ (Le Genest, France). Postnatal day 8 pups were studied.
Wild animals	Wild animals were not used.
Field-collected samples	The study did not involve field collected samples.

Ethics oversight	All animal research was performed under the EU adopted Directive 2010/63/EU and reviewed by the local Comité National de Réflexion Ethique sur l'Expérimentation animale.

Note that full information on the approval of the study protocol must also be provided in the manuscript.

Human research participants

Policy information about studies involving human research participants

Population characteristics	Term neonates > 36 weeks' gestation were studied. Based on their clinical characteristics, they were grouped into those with moderate to severe encephalopathy and those with mild encephalopathy. The details of the participants are provided in Table 1.
Recruitment	Consecutive neonates recruited in the biomarker study were included in this, if adequate samples were available.
Ethics oversight	The study was approved by a UK research ethics committee, NRES Committee London-Bromley (REC ref:13/LO/1738).

Note that full information on the approval of the study protocol must also be provided in the manuscript.
